# Nutritional valorization of Manila tamarind accessions through antioxidant analysis and UHPLC-Q-TOF-MS-based metabolomic profiling

**DOI:** 10.3389/fnut.2025.1646522

**Published:** 2025-09-02

**Authors:** Ashok Yadav, Suchisree Jha, Girija Choudhary, Asha Ram, Rajeev Kumar, Naresh Kumar, Hirdayesh Anuragi, Sandeep Garg, Anil Kumar, Pradyuman Singh, Raghunandan Prasad Dwivedi, Ayyanadar Arunachalam, Dinesh Jinger

**Affiliations:** ^1^ICAR-Central Agroforestry Research Institute, Jhansi, Uttar Pradesh, India; ^2^R and D, Indofil Industry Ltd., Thane West, Maharashtra, India; ^3^Bundelkhand University, Jhansi, Uttar Pradesh, India; ^4^ICAR-Indian Institute of Soil and Water Conservation, Regional Center-Chandigarh, Chandigarh, India

**Keywords:** functional foods, Manila tamarind, nutraceuticals, natural antioxidants underutilized, UHPLC-Q-TOF-MS

## Abstract

**Introduction:**

Manila tamarind is an underutilized and multipurpose crop with considerable value for food, fodder, fuel, and green manuring. Its hardy nature, drought tolerance, and diverse applications make it a promising climate-smart crop for agroforestry systems, especially in the drylands of the Bundelkhand region. However, research on its antioxidant potential and phytochemical composition has been largely neglected.

**Methods:**

To address this gap, the present study was conducted to assess the antioxidant content and identify health-related phytochemicals in the arils of 15 different Manila tamarind accessions, which were collected locally from the Bundelkhand region. Antioxidant analysis (DDPH, ABTS, Metal Chelating), phenol, flavonol, and anthocyanin were estimated as per standard procedures, whereas the phytochemicals were estimated through UHPLC-Q-TOF-MS analysis.

**Results:**

The results revealed significant variation in vitamin content (vitamin A: 0.18–0.28 mg/100 g, thiamin: 0.18–0.24 mg/100 g, riboflavin: 0.11–0.18 mg/100 g, vitamin C: 122–139 mg/100 g, and b-carotenoid equivalent 11.4–19.6 mg/100 g) and antioxidant activity across various assays, including DPPH (65.58–282.44 mg/ml), ABTS (117.80–508 IC50 mg/ml), metal chelating capacity (167.41–376.06 IC50 mg/ml), total phenolic content (0.019–0.174 mg GAE/g fresh weight), and total flavonol content (0.0042–0.0088 mg QE/g fresh weight). Anthocyanin levels ranged from 0.126 to 0.262 mg TAC/g fresh weight. Phytochemical profiling led to the identification of 144 compounds, which were classified into 43 biological function categories. The predominant compounds exhibited antioxidant, anti-inflammatory, anticancer, neuroprotective, antifungal, antibacterial, antimicrobial, antiviral, anti-tumor, analgesic, cardioprotective, and antidiabetic properties, highlighting the crop's immense potential for health and nutritional applications.

**Conclusion:**

The primary aim of this study was to evaluate the potential of this plant, and the findings provide strong evidence that this plant possesses significant bioactive compounds, suggesting its potential use in combating a range of infectious diseases. In addition to this, the findings of this study are valuable for selecting superior parent lines to enhance desirable traits in future Manila tamarind breeding programs.

## 1 Introduction

Manila tamarind (*Pithecellobium dulce*), also known as sweet tamarind, is a member of the Leguminosae family and belongs to the genus Pithecellobium, which comprises 18 species. This versatile tree is known by various names across different languages and regions, reflecting its wide distribution and cultural significance. Some of these are known by various names in different languages, such as Monkey Pod (English), Vilayati Imli, Jangal Jalebi, Singri, Dakhani Babul (Hindi), Kodukkappuli (Tamil), Vilayati Chinch (Marathi), Me Keo, Keo Tay (Vietnamese), Asam Koranji (Indonesian), and Makham Thet (Thai). Manila tamarind is a fast-growing, underutilized tree species that is hardy, evergreen, highly drought-tolerant, offers significant nutritional benefits, and plays a vital role in improving food security and reducing poverty among communities living in challenging agro-climatic conditions (1). It is remarkably adaptable and capable of thriving in nutritionally poor and environmentally challenging conditions. The species grows well across a wide range of soil types, including sandy, loamy, and clayey soils, as well as acidic, neutral, alkaline, and even saline environments (2). Manila tamarind can grow at elevations up to 1,550 meters and withstand extreme temperatures, tolerating conditions as high as 48 °C in arid, hot tropical, and subtropical regions. Its adaptability also extends to varying rainfall regimes—it thrives in areas receiving annual precipitation between 700 and 1,800 mm and can survive in regions with as little as 250 mm of rainfall (2, 3). The seeds and pods of Manila tamarind are highly nutritious, with the aril, edible pulp, being particularly rich in essential nutrients. It contains moisture (75.8–77.8 g), energy (78.8 kcal), ash (0.6%), protein (12.47–23.3 g), fat (0.4–0.5 g), carbohydrates (18.2–76.87 g), fiber (1.1–1.3 g), calcium (13–21 mg), phosphorus (42–58 mg), iron (0.5–1.1 mg), sodium (3.7–19 mg), potassium (222–377 mg), magnesium (40 mg), and copper (13.8–33.0 mg) per 100 g of aril (4–6). As a leguminous species, Manila tamarind has nitrogen-fixing properties that enhance soil fertility. Its fast growth, spiny structure, and dense branching make it an excellent choice for use as a bio-fence against wild and stray animals. Additionally, the wood is valued for furniture-making and tool construction, serves as a good source of firewood due to its high calorific value, and is widely employed in windbreaks and shelterbelts (7).

Since ancient times, human civilizations have relied on plants for the development of therapeutic agents. The traditional use of natural products in treating various ailments underscores the importance of exploring plant-based sources for novel pharmacological compounds (8). The therapeutic potential of medicinal plants is primarily attributed to the diversity and complexity of their phytochemical constituents, which exert a wide range of physiological effects on the human body (9). Consequently, phytochemical screening plays a crucial role in identifying these bioactive compounds, laying the groundwork for the discovery and development of modern medicines (10).

There has been a significant rise in microbial resistance to synthetic drugs, coupled with a decline in the development of new antimicrobial agents (3). In response to this growing challenge, attention has increasingly shifted toward the discovery of novel, effective, and affordable therapeutic alternatives, particularly for combating microbial infections prevalent in underdeveloped and developing countries, where infectious diseases account for nearly 50% of the mortality rate (3). Phytochemicals, the secondary metabolites produced through diverse plant metabolic pathways, have emerged as promising candidates for drug development due to their potent antimicrobial properties and natural origin. In light of the escalating threat posed by multidrug-resistant microbes, there is a growing emphasis in modern medicine on the urgent need to identify and develop innovative phytochemicals from natural sources. These compounds hold significant potential to provide effective and sustainable solutions to combat antimicrobial resistance and improve human health outcomes.

Furthermore, cancer remains a leading global health challenge, with ~20 million new cases and 10 million cancer-related deaths recorded in 2020 alone (4). The most common cancer types include lung, breast, colorectal, prostate, and gastric cancers. This is projected to increase by 47% by 2040, particularly in low- and middle-income countries, due to demographic shifts, urbanization, and limited access to early diagnosis and preventive healthcare (5). Despite the traditional use of Manila tamarind for its nutritional and therapeutic properties, there is a notable lack of comprehensive scientific studies focusing on the antioxidant capacity, phytochemical composition, anthocyanin concentration, and vitamin content of its aril. While various plant-based foods have been extensively analyzed for their bioactive compounds, Manila tamarind remains underexplored, particularly for its potential health-promoting properties using modern analytical methodologies. This gap hinders the full utilization and recognition of the species in functional food and nutraceutical development. Hence, identifying accessions with superior yield and biochemical traits forms the foundation for developing high-yielding, nutritionally enhanced cultivars (6–8). Therefore, the present investigation aims to systematically evaluate its antioxidant activity, anthocyanin content, vitamin profile, and overall phytochemical composition using advanced analytical techniques.

## 2 Materials and methods

The field survey for the collection of different Manila tamarind accessions was carried out in the three villages (Bhojla, Karari, and Simardha) of Jhansi district of Uttar Pradesh. Jhansi is the heart of the Bundelkhand region (23°10′-26°30′N and 78°20′-81°40′E), which has a semi-arid type climate with a moisture deficiency index varying from 40 to 60. A total of 15 Manila tamarind (MT) accessions (MT-1 to MT-15) were collected, and dried Manila tamarind pods were initially cleaned with tap water, followed by a rinse with distilled water to remove surface impurities. The pods were then air-dried at room temperature. After drying, the pods were manually crushed to separate the aril from the seeds. The aril (seed-free pulp) was ground into a coarse powder using a mortar and pestle. The resulting powder was stored in sealed containers under dry conditions and later used for further nutraceutical analyses, including antioxidant activity, mineral content, and vitamin composition.

### 2.1 Sample extraction

Methanolic extracts of Manila tamarind aril were prepared using the cold maceration technique, as described by Omaye et al. (11). Precisely, 50 g of coarsely powdered aril was placed in stoppered containers containing 250 mL of methanol. The mixture was kept at room temperature for 72 h with frequent shaking to facilitate the extraction of soluble phytochemicals. Following maceration, the mixture was filtered through Whatman No. 1 filter paper (125 mm) to obtain a clear filtrate. The filtrate was then concentrated using a rotary evaporator—aqueous extracts at 100 °C, and methanolic extracts at 78 °C—until the volume was reduced to one-fourth of the original. The concentrated extracts were reconstituted in an appropriate volume of solvent to achieve the desired concentration and stored in desiccators until further use.

### 2.2 Antioxidant analysis

Antioxidant analysis of 15 Manila tamarind accessions was conducted using three different methods: DPPH (2,2-Diphenyl-1-picrylhydrazyl) (12, 13), ABTS (22′-azino-bis(3-ethylbenzothiazoline-6-sulfonic acid)) (14), and metal chelating activity (MCA) (15). The results of DPPH, ABTS, and metal chelating assays were expressed in terms of IC50 (mg/ml), indicating the concentration required to inhibit 50% of free radicals or metal ions.

### 2.3 Phenol, flavonol, and anthocyanin content

Total phenolic and flavonol contents were measured as TPC (mg/g fresh weight tissue, FWT), while anthocyanin content (16) was expressed as total anthocyanin content (TAC, mg/g FWT). Among the Manila tamarind accession, total phenolic content (TPC) (17) and total flavonol content (TFC) (18) were calculated as per the standard method. Anthocyanin content in Manila tamarind samples was estimated using the pH differential method, as described by Wallace and Giusti (16). In this technique, aril samples are first homogenized and extracted with an acidified solvent—typically methanol or ethanol, containing 0.1% hydrochloric acid (HCl)—to stabilize the anthocyanins. The extract is then filtered or centrifuged to remove any solid residues. Two aliquots of the clarified extract are prepared: one is diluted with a buffer at pH 1.0, and the other with a buffer at pH 4.5.

Absorbance readings are recorded at 520 nm, where anthocyanins exhibit peak absorbance, and at 700 nm to correct for haze or turbidity, using a UV-vis spectrophotometer. The difference in absorbance between the two pH conditions is used to calculate anthocyanin concentration. Results are typically expressed as cyanidin-3-glucoside equivalents (mg/g fresh weight), based on a standard formula that incorporates molecular weight, molar extinction coefficient, path length, and dilution factors.

The absorbance (A) is calculated using the following formula:


$$\begin{array}{l} A={\left(A520-A700\right)}_{\text{pH }1.0}-{\left(A520-A700\right)}_{\text{pH }4.5} \end{array}$$

The monomeric anthocyanin pigment concentration (expressed as cyanidin-3-glucoside equivalents) is then calculated by:


$$\begin{array}{l} Anthocyanin\;content\;\left(mg/L\right)=A\;\times \;MW\;\times \;DF\;\times \;1,000/\varepsilon xl \end{array}$$

where:

- **MW** = molecular weight of cyanidin-3-glucoside (449.2 g/mol),
- **DF** = dilution factor,
- **ε** = molar extinction coefficient (26,900 L·mol^−1^·cm^−1^ for cyanidin-3-glucoside),
- **l** = path length of cuvette (1 cm).

### 2.4 Vitamin content analysis

Fresh and ripe pods of Manila tamarind were collected to estimate different vitamin contents, including vitamin A, thiamin, riboflavin, vitamin C, and β-carotenoid equivalents. The values were expressed in mg/100 g. The details of the methodology are described in the following sections.

#### 2.4.1 Vitamin A

The vitamin-A content was estimated among Manila tamarind accessions using a colorimetric method as per the method suggested by Rodriguez-Amaya and Kimura (9). The sample is first saponified using alcoholic potassium hydroxide (KOH) to release retinol from esterified forms. The unsaponifiable matter, including retinol, is extracted using petroleum ether. After evaporation and redissolution in chloroform, trichloroacetic acid (TCA) is added, leading to the development of a blue color that is measured at 620 nm using a spectrophotometer (9).

#### 2.4.2 Thiamin (vitamin B_1_)

Thiamin in Manila tamarind accessions was estimated using the fluorometric thiochrome method (10). The sample undergoes acid hydrolysis with 0.1N hydrochloric acid, followed by enzymatic digestion, often using takadiastase, to release thiamin. It is then oxidized with alkaline potassium ferricyanide to form thiochrome, a fluorescent compound. Thiochrome is extracted into isobutanol, and its fluorescence is measured with excitation at 366 nm and emission at 435 nm.

#### 2.4.3 Riboflavin (vitamin B_2_)

Riboflavin in Manila tamarind accessions was estimated using a fluorometric method that involved both acid hydrolysis and enzymatic digestion for vitamin extraction, followed by fluorometric analysis (19). Approximately 5 g of the sample were homogenized and hydrolyzed with 50 mL of 0.1 N hydrochloric acid by heating in a boiling water bath for 30 min to break protein–vitamin complexes and release bound riboflavin. After cooling, the pH was adjusted to around 4.5 using sodium acetate buffer, and enzymatic digestion was carried out by adding takadiastase enzyme, allowing the mixture to incubate at 37 °C for 2 h to further liberate riboflavin. Following digestion, the mixture was filtered, and the filtrate was subjected to fluorometric analysis. Riboflavin's natural fluorescence was measured using a fluorometer set at an excitation wavelength of 450 nm and an emission wavelength of 520 nm. Quantification was done by comparing sample fluorescence to a standard riboflavin curve prepared under identical conditions.

#### 2.4.4 Vitamin C

The estimation of vitamin C (ascorbic acid) was performed using the 2,6-dichlorophenolindophenol (DCPIP) titration method (11). In this method, approximately 5 g of the sample was homogenized in 50 mL of a 3% metaphosphoric acid solution, which serves to precipitate proteins and prevent oxidative degradation of ascorbic acid by stabilizing it in the acidic medium. The homogenate is filtered through Whatman No. 1 filter paper to obtain a clear extract. An aliquot (usually 10 ml) of the filtrate is then titrated against a freshly prepared standard DCPIP dye solution of known concentration. The dye is reduced by ascorbic acid, leading to a color change from blue to colorless; the endpoint of the titration is marked by the appearance of a light pink color that persists for at least 15 s, indicating that all the ascorbic acid has been oxidized. The amount of DCPIP used is directly proportional to the amount of vitamin C in the sample. A standard curve using known concentrations of ascorbic acid is used for calibration to calculate the vitamin C content, usually expressed in mg per 100 g of sample.

#### 2.4.5 β-carotene equivalents estimation

The β-carotene equivalents in Manila tamarind samples were estimated using a colorimetric method involving extraction with organic solvents followed by spectrophotometric analysis, as described by Rodriguez-Amaya and Kimura (9). In this method, approximately 5 g of a finely homogenized sample was extracted with cold acetone to solubilize carotenoids, including β-carotene. The extraction is carried out under low-light conditions to prevent degradation, and the extract is filtered through filter paper. The acetone extract is then transferred to a separating funnel containing petroleum ether (or hexane), and the carotenoids are partitioned into the non-polar solvent. The aqueous phase is discarded, and the ether layer is washed several times with distilled water to remove any residual acetone and polar impurities. The ether phase, now containing the β-carotene, is collected, and its absorbance is measured at 450 nm using a UV–vis spectrophotometer. The β-carotene content is then quantified by comparing the absorbance to a standard calibration curve prepared with known concentrations of pure β-carotene, and results are expressed as β-carotene equivalents (mg/100 g sample).

### 2.5 Phytochemical profiling of Manila tamarind accessions

For the phytochemical profiling of Manila tamarind (*Pithecellobium dulce*), samples were analyzed using a Vanquish UHPLC (ultra-high-performance liquid chromatography) system coupled with a Q Exactive™ quadrupole-Orbitrap mass spectrometer (Thermo Fisher Scientific) on the IITB_QE-PC platform. Extracts were filtered through a 0.22 μm membrane filter before injection. Separation was carried out on a Hypersil GOLD C18 column (100 × 2.1 mm, 1.9 μm particle size) maintained at 40 °C. The mobile phases used were (A) 0.1% formic acid in water and (B) 0.1% formic acid in acetonitrile. A gradient elution was employed as follows: 0–2 min: 5% B; 2–20 min: 5% B; 20–25 min: linear increase to 95% B; 25–26 min: hold at 95% B; 26–30 min: 5% B; and 30–35 min: re-equilibrate to 5% B. The flow rate was maintained at 3 μL/min, and the injected sample volume was 5 μL. The highest flow rate for ramp-up and down was set at 6.0 ml/min^2^, with pressure ranging from 0 to 1,034 bar. The mass spectrometer operated in positive electrospray ionization (ESI+) mode with the following parameters: spray voltage 3.5 kV, capillary temperature 320 °C, sheath gas flow 35 units, auxiliary gas flow 10 units, and S-lens RF level 50. Full scan MS data were acquired over an m/z range of 100–1,000 at a resolution of 70,000 (FWHM at m/z 200) with an automatic gain control (AGC) target of 1e6. Data-dependent MS/MS acquisition was enabled to fragment the top 5 most intense ions per scan cycle. The identification process comprised several critical steps, including library matching, feature recognition, elemental composition analysis, background subtraction using blank samples, retention time alignment, and fragmentation search (FISh) scoring with a threshold above 40. Phytochemicals present in the aril of Manila tamarind were primarily identified by comparing MS/MS spectra with the mzCloud database, while unmatched signals were further analyzed and cross-referenced using the ChemSpider database for confirmation (12).

### 2.6 Statistical analysis

The data on antioxidants, anthocyanins, and vitamins of 15 Manila tamarind accessions were analyzed as per the procedure of the analysis of variance (ANOVA) for the completely randomized block design (CRBD), and significance was tested at 5% level (13). The differences between mean values were also determined using Duncan's multiple range test at a 5% significance level.

## 3 Results and discussion

### 3.1 Antioxidant analysis

Antioxidants are vital to human health as they neutralize free radicals—unstable molecules that can damage cells, proteins, and DNA through oxidative stress. By mitigating this damage, antioxidants help reduce the risk of chronic conditions, including cardiovascular disease, cancer, diabetes, and neurodegenerative disorders such as Alzheimer's disease. Antioxidants also support immune function, reduce inflammation, and slow down the aging process by preserving skin and tissue health (14). Considering this, we estimated the antioxidant activity of 15 Manila tamarind accessions using multiple assays, including DPPH, ABTS, metal chelating activity, total phenolic content, and flavonol content. All the results were statistically significant (*p* < 0.05) and are summarized in Table 1. The DPPH radical scavenging activity (IC50) ranged from 65.58 mg/g (MT-5) to 282.44 mg/g (MT-10), with an average of 117.91 mg/g, indicating a broad spectrum of antioxidant capacities of these accessions. Significantly lower IC50 values of MT-5 suggested stronger antioxidant activity and higher potency. Similarly, ABTS assay exhibited maximum antioxidant activity in MT-14 (117.80 IC_50_ mg/g) while minimum in MT-9 (508.94 IC_50_ mg/g) with an overall mean of 237.17 IC_50_ mg/g. The MC assay unveiled MT-2 with a significantly higher value (167.41 IC_50_ mg/g), followed by MT-15 (200.13 IC_50_ mg/g) and MT-13 with the lowest (376.06 IC_50_ mg/g) antioxidant activity. The study showed that Manila tamarind has the ability to chelate metals, which may function as a protective mechanism against oxidative damage brought on by metal-catalyzed degradation processes (15).

**Table 1:** Antioxidant and anthocyanin analysis of different Manila tamarind accessions.

**Accessions**	**DPPH (IC50 mg/g)**	**ABTS (IC50 mg/g)**	**Metal chelating (IC50 mg/g)**	**Phenol (TPC GAE/g FWT)**	**Flavonol (TFC QE/g FWT)**	**Anthocyanin (TAC/g FWT)**
MT-1	95.27 ± 5.05^fgh^	190.97 ± 10.13^fg^	240.71 ± 12.76^f^	0.090 ± 0.0048^de^	0.0069 ± 0.00037^c^	0.16 ± 0.01^f^
MT-2	93.91 ± 3.04^fgh^	315.51 ± 10.21^d^	167.41 ± 5.42^h^	0.099 ± 0.0032^bc^	0.0088 ± 0.00028^a^	0.19 ± 0.01^e^
MT-3	121.29 ± 2.73^c^	169.52 ± 3.82^hi^	355.77 ± 8.01^b^	0.0104 ± 0.0023^b^	0.0060 ± 0.00014^d^	0.21 ± 0.00^d^
MT-4	89.41 ± 3.38^h^	189.46 ± 7.15^fg^	238.37 ± 9.00^f^	0.0940 ± 0.0035^cd^	0.0078 ± 0.00029^b^	0.20 ± 0.01^de^
MT-5	65.58 ± 3.85^i^	194.76 ± 11.42^f^	227.88 ± 13.37^f^	0.0950 ± 0.0056^cd^	0.0073 ± 0.00043^c^	0.18 ± 0.01^e^
MT-6	99.52 ± 4.08^efg^	201.04 ± 8.23^ef^	331.01 ± 13.56^cd^	0.0820 ± 0.0034^f^	0.0060 ± 0.00025^d^	0.13 ± 0.01^g^
MT-7	101.30 ± 2.28^ef^	193.93 ± 4.37^f^	270.16 ± 6.08^e^	0.0840 ± 0.0019^ef^	0.0048 ± 0.00011^f^	0.25 ± 0.01^abc^
MT-8	99.82 ± 5.86^efg^	212.03 ± 12.44^e^	328.75 ± 19.28^cd^	0.0850 ± 0.0050^ef^	0.0053 ± 0.00031^e^	0.26 ± 0.02^abc^
MT-9	110.40 ± 5.11^d^	508.94 ± 23.57^a^	336.92 ± 15.60^bcd^	0.0800 ± 0.0037^f^	0.0045 ± 0.00021^fg^	0.26 ± 0.01^a^
MT-10	282.44 ± 11.87^a^	188.64 ± 7.93^fg^	345.22 ± 14.51^bc^	0.0890 ± 0.0037^de^	0.0049 ± 0.00021^ef^	0.26 ± 0.01^ab^
MT-11	102.04 ± 4.16^def^	338.16 ± 13.77^c^	269.02 ± 10.96^e^	0.0890 ± 0.0036^de^	0.0082 ± 0.00033^b^	0.25 ± 0.01^abc^
MT-12	104.41 ± 1.19^de^	404.31 ± 4.59^b^	234.00 ± 2.66^f^	0.0200 ± 0.0009^f^	0.0053 ± 0.00006^e^	0.24 ± 0.00^c^
MT-13	94.64 ± 3.88^fgh^	176.49 ± 7.23^gh^	376.06 ± 15.40^a^	0.0800 ± 0.0033^f^	0.0042 ± 0.00017^g^	0.25 ± 0.01^bc^
MT-14	92.17 ± 1.25^gh^	117.80 ± 1.59^j^	321.17 ± 4.34^d^	0.0190 ± 0.0003^g^	0.0043 ± 0.00006^g^	0.25 ± 0.00^abc^
MT-15	216.39 ± 8.81^b^	155.92 ± 6.35^i^	200.13 ± 8.15^g^	0.0174 ± 0.0071^a^	0.0060 ± 0.00024^d^	0.26 ± 0.01^abc^
F stat	361.20^**^	350.59^**^	93.78^**^	195.49^**^	105.91^**^	78.79^**^
SEm ±	2.95	5.70	6.554	0.0022	0.00014	0.005
LSD (*P* = 0.05)	8.55	16.52	18.98	0.0063	0.00041	0.014

Values followed by letter in common within column indicate no significant difference among the treatments (*p* = 0.05). ^**^ indicates significance at 1% level.

### 3.2 Phenol, flavonol, and anthocyanin content

Manila tamarind accessions exhibited a low range of phenol and flavonol content in the arils of the pods. Table 1 shows the values of total phenolic content (TPC) ranging from 0.019 mg Gallic Acid Equivalent (GAE)/g FWT (MT-14) to 0.174 mg GAE/g FWT (MT-12), with a mean value of 0.089 mg GAE/g FWT. The total flavonol content (TFC) ranged between 0.0042 mg QE/g FWT (MT-13) and 0.0088 mg QE/g FWT (MT-2), with a mean of 0.0060 mg QE/g FWT. Phenolic compounds are well-documented for their therapeutic potential in managing various human health disorders, including hypertension, metabolic syndromes, inflammatory conditions, and neurodegenerative diseases. Their efficacy is primarily attributed to their ability to inhibit key enzymes involved in the progression of these conditions (17). Among them, flavonoids—a major subclass of phenolics—demonstrate a broad spectrum of biological activities, such as antiviral, anticancer, antioxidant, and anti-inflammatory effects. Additionally, they possess cardioprotective and neuroprotective properties, contributing significantly to disease prevention and overall health maintenance (18). Consequently, extensive screening of additional Manila tamarind accessions is warranted to identify genotypes with superior phenol and flavonol content. Such efforts could support the development of functional foods aimed at reducing disease incidence in human populations. The findings of the present study highlight the considerable phytochemical and antioxidant diversity among Manila tamarind accessions, offering valuable potential for breeding programs focused on enhancing nutritional and therapeutic value. Anthocyanins and other dietary bioactive compounds contribute significantly to long-term health and wellbeing through their diverse biological activities. Regular consumption of colorful fruits and vegetables, rich in natural sources of these compounds, is an essential component of a balanced diet and has been associated with a reduced risk of various chronic diseases (16). Total anthocyanin content (TAC) in different Manila tamarind accessions (Table 1) indicated anthocyanin content ranged from 0.126 (MT-6) to 0.262 TAC mg/g FWT (MT-9), with a mean of 0.22 mg/g FWT. Superior accessions such as MT-9 and MT-10, with higher TAC, may serve as promising candidates for nutraceutical applications (20).

### 3.3 Vitamins and pigments

Vitamin profiling of 15 Manila tamarind accessions revealed considerable variability in the content of vitamin A (mg/100 g), thiamin (mg/100 g), riboflavin (mg/100 g), vitamin C (mg/100 g), and β-Carotenoid equivalents (mg/100 g) (Table 2). The vitamin A ranged from 0.18 (MT-6) to 0.28 mg/100 g (MT-12), thiamin ranged from 0.18 mg (MT-5 and MT-12) to 0.24 mg (MT-10), riboflavin ranged from 0.11 mg (MT-1, MT-14) and 0.18 mg (MT-2), vitamin C from 122 mg (MT-14) to 139 mg (MT-9), and β-carotenoid equivalents ranged from 11.4 mg (MT-1) to 19.6 mg (MT-9).

**Table 2:** Vitamin content in pods of different Manila tamarind accessions.

**Accessions**	**Vitamin A (mg/100 g)**	**Thiamin (mg/100 g)**	**Riboflavin (mg/100 g)**	**Vitamin C (mg/100 g)**	**β-carotenoid equivalent (mg/100 g)**
MT-1	0.22 ± 0.01^e^	0.19 ± 0.01^ef^	0.11 ± 0.01^h^	129.00 ± 6.84^bcde^	11.40 ± 0.47^h^
MT-2	0.24 ± 0.01^d^	0.22 ± 0.00^bc^	0.18 ± 0.01^a^	131.00 ± 4.24^abcd^	14.20 ± 0.32^ef^
MT-3	0.21 ± 0.00^ef^	0.21 ± 0.01^cd^	0.15 ± 0.00^d^	136.00 ± 3.06^ab^	17.10 ± 1.00^b^
MT-4	0.19 ± 0.01^gh^	0.20 ± 0.01^de^	0.14 ± 0.01^e^	133.00 ± 5.02^abc^	15.70 ± 0.73^cd^
MT-5	0.21 ± 0.01^ef^	0.18 ± 0.01^f^	0.16 ± 0.01^c^	134.00 ± 7.86^abc^	13.20 ± 0.55^fg^
MT-6	0.18 ± 0.01^h^	0.22 ± 0.01^bc^	0.13 ± 0.01^f^	133.00 ± 5.45^abc^	12.40 ± 0.51^gh^
MT-7	0.20 ± 0.00^fg^	0.20 ± 0.00^de^	0.14 ± 0.00^e^	131.00 ± 2.95^abcd^	16.10 ± 0.18^bcd^
MT-8	0.24 ± 0.01^d^	0.20 ± 0.01^de^	0.13 ± 0.01^f^	130.00 ± 7.63^bcde^	16.40 ± 0.67^bc^
MT-9	0.27 ± 0.01^ab^	0.21 ± 0.00^cd^	0.17 ± 0.01^b^	139.00 ± 6.44^a^	19.60 ± 0.27^a^
MT-10	0.22 ± 0.01^e^	0.24 ± 0.01^a^	0.14 ± 0.01^e^	127.00 ± 5.34^cde^	12.30 ± 0.65^gh^
MT-11	0.26 ± 0.01^bc^	0.23 ± 0.01^ab^	0.12 ± 0.00^g^	124.00 ± 5.05^de^	15.20 ± 0.49^de^
MT-12	0.28 ± 0.00^a^	0.18 ± 0.00^f^	0.15 ± 0.00^d^	137.00 ± 1.56^ab^	13.70 ± 0.31^f^
MT-13	0.25 ± 0.01^cd^	0.21 ± 0.01^cd^	0.12 ± 0.00^g^	126.00 ± 5.16^cde^	16.20 ± 0.61^bcd^
MT-14	0.21 ± 0.00^ef^	0.19 ± 0.01^ef^	0.11 ± 0.00^h^	122.00 ± 1.65^e^	15.60 ± 0.92^cd^
MT-15	0.26 ± 0.01^bc^	0.21 ± 0.01^cd^	0.16 ± 0.01^c^	129.00 ± 5.25^bcde^	16.30 ± 0.67^bc^
F stat	34.37^**^	12.79^**^	44.23^**^	2.64^*^	38.92^**^
SEm±	0.005	0.005	0.003	2.94	0.34
LSD (*P = 0.05*)	0.015	0.014	0.009	8.52	1.00

Values followed by letter in common within column indicate no significant difference among the treatments (*p* = 0.05). ^*^ indicates significance at 5% level. ^**^ indicates significance at 1% level.

The highest vitamin A content of accession MT-12 indicated its potential therapeutic use in supporting vision and immune functions. These values align with previous studies indicating that legumes and tropical fruits are effective sources of vitamin A (21). Thiamin, essential for carbohydrate metabolism, was found in appreciable amounts in accessions MT-10, MT-11, and MT-2, suggesting their potential utility in dietary interventions aimed at improving thiamin intake. Riboflavin, which plays a key role in energy production and cellular function (22), showed relatively low variability across accessions, indicating possible genetic stability of this trait. Vitamin C, a potent antioxidant essential for collagen synthesis and immune defense, was present in significant quantities. With a daily recommended intake of 90 mg for men and 75 mg for women, the vitamin C content in Manila tamarind was approximately 1.6 times higher than the recommended daily allowance, suggesting that regular consumption could readily meet and exceed daily requirements. The observed vitamin C levels were notably higher than those reported for many commonly consumed fruits, positioning Manila tamarind as a rich natural source of ascorbic acid (23). Similarly, β-carotenoids, which serve both as precursors to vitamin A and as antioxidants (24), were found in higher concentrations in accessions MT-9, MT-3, and MT-8. Given the recommended daily intake of β-carotene (6–15 mg/day for adults and adolescents), the required amount can be fulfilled by consuming approximately 80–90 g of Manila tamarind. Among all accessions, MT-9 exhibited the highest levels of both vitamin C and β-carotenoids. Accessions MT-9, MT-12, and MT-2 consistently recorded elevated levels of multiple vitamins and carotenoids, marking them as promising candidates for use in nutritional improvement programs and the development of functional foods.

### 3.4 Phytochemical profiling

**Figure 1:**
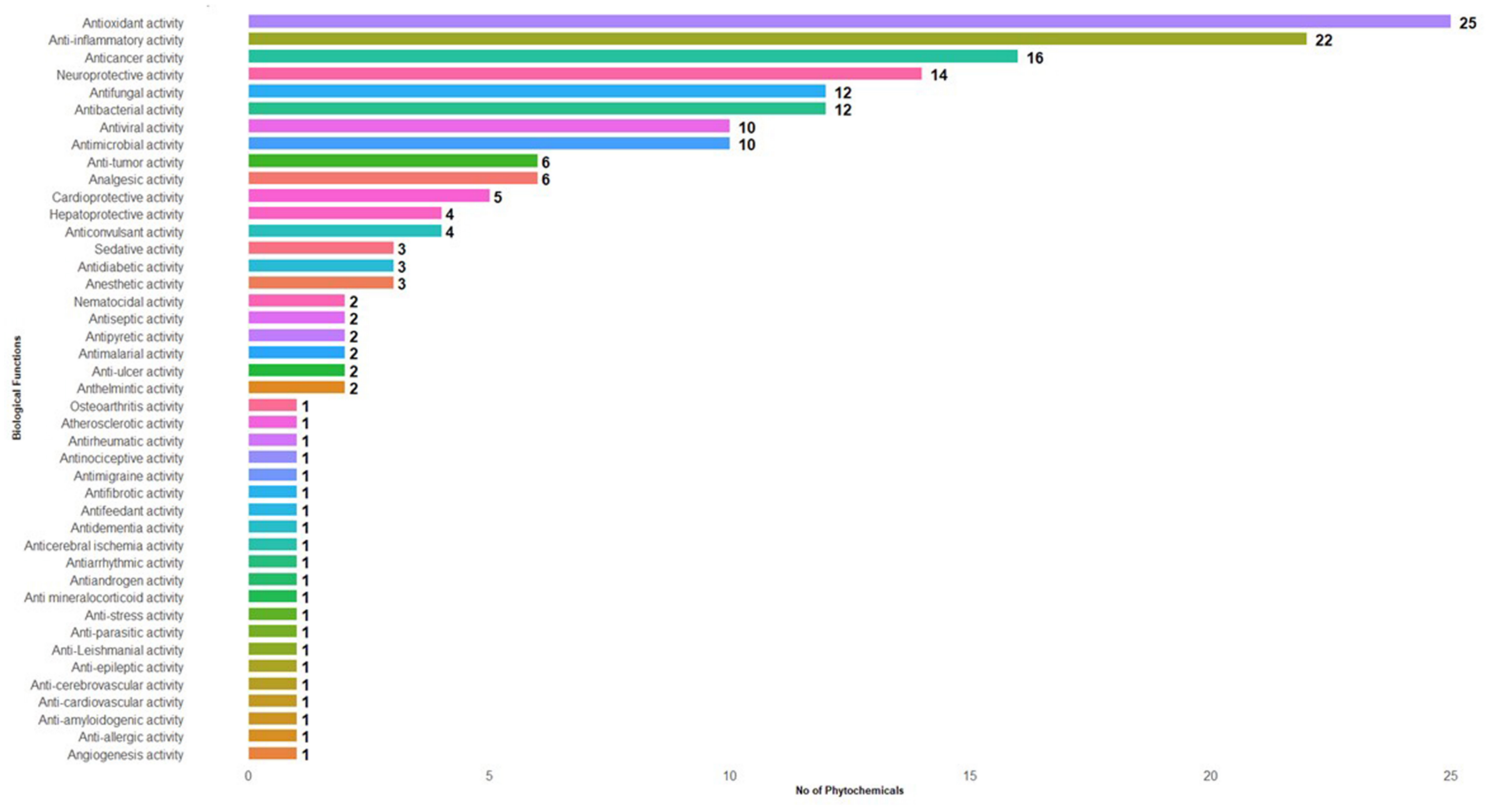
List of biological functions and the number of phytochemical compounds found in the aril of Mania tamarind.

**Figure 2:**
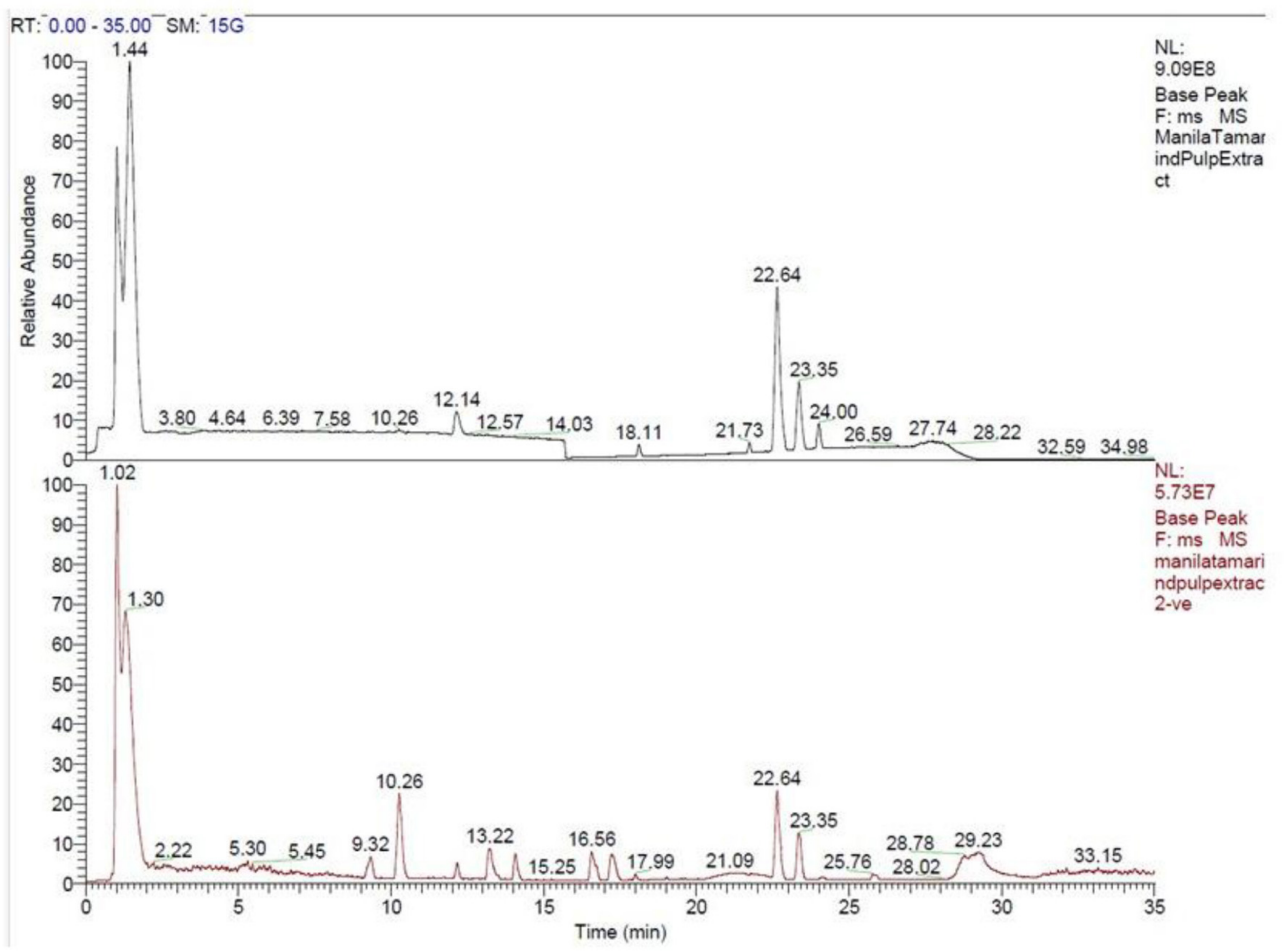
Total ion chromatograms (TIC) of methanolic extract of Manila tamarind pulp in positive (**top**, black) and negative (**bottom**, red) ionization modes obtained via LC-MS analysis. The x-axis represents retention time (RT, in minutes) while the y-axis shows relative abundance of ion signals. The presence of the prominent peaks indicates the presence of multiple phytoconstituents in both modes.

A total of 144 phytochemicals were identified in Manila tamarind, comprising 117 compounds detected in positive ionization mode and 27 in negative ionization mode. These phytochemicals were functionally classified based on their biological activities and grouped into 43 distinct categories (Figure 1, Table 3). LC-MS chromatographic profiling of the methanolic pulp extract revealed a diverse and complex phytochemical landscape. The total ion chromatogram (TIC) demonstrated elution of various compounds within a 0–35 min retention time (RT) window (Figure 2). The positive ionization mode spectrum exhibited the prominent peaks at RT 1.44, 3.80, 6.39, 10.26, 12.14, and a cluster of intense peaks between 22.64 and 24.00 min. In contrast, negative ion mode revealed sharper and more intense peaks at RT 1.30, 10.26, 13.22, 15.26, and 22.64 min. Notably, a strong and consistent peak at RT 22.64 min was observed in both modes, suggesting the presence of a key compound detectable across polarities, indicative of its abundance or unique ionization behavior.

**Table 3:** Phytochemicals identified in Manila tamarind through the positive and negative mode of analysis.

**Name**	**Formula**	**Annot. delta mass [ppm]**	**Calc. MW**	**RT [min]**	**mz cloud best match**
**Phytochemicals identified through the positive mode of analysis**					
(-)-Fustin	C_15_H_12_O_6_	−0.18	288.06	9.29	65.90
(±)-Abscisic acid	C_15_ H_20_O_4_	−0.12	264.14	13.49	89.60
(2E)-3-(4-{[(2S,3R,4S,5S,6R)-3,4,5-trihydroxy-6-(hydroxymethyl)oxan-2-yl]oxy}phenyl)prop-2-enoic acid	C_15_H_18_O_8_	−0.35	326.10	9.26	61.70
(2S6′R)2′,4,6-trimethoxy6′-methyl-3H-spiro[1-benzofuran-21′-cyclohexan]2′-ene-34′-dione	C_17_H_18_O_6_	−0.10	318.11	14.71	96.00
[(3R,4S)-1-(4-Morpholinylcarbonyl)-3-(2-{4-[3-(trifluoromethyl)phenyl]-1-piperazinyl}ethyl)-4-piperidinyl]acetic acid	C_25_H_35_F_3_N_4_O_4_	−2.51	512.26	15.44	91.10
1-(2-Morpholinophenyl)dihydro-1H-pyrrole-2,5-dione	C_14_H_16_N_2_O_3_	0.29	260.12	7.78	66.20
1-(3,4-dimethoxyphenyl)ethan-1-one oxime	C_10_H_13_NO_3_	0.69	195.09	3.21	68.60
1-Stearoylglycerol	C_21_H_42_O_4_	−0.54	358.31	23.34	98.70
2-(3,4-dihydroxyphenyl)-5,7-dihydroxy-3-{[(2S,3R,4R,5R,6S)-3,4,5-trihydroxy-6-methyloxan-2-yl]oxy}-4H-chromen-4-one	C_21_H_20_O_11_	0.09	448.10	13.23	96.70
2,2,6,6-Tetramethyl-1-piperidinol (TEMPO)	C_9_H_19_NO	0.32	157.15	15.64	40.00
2,3-dihydroxypropyl 12-methyltridecanoate	C_17_H_34_O_4_	0.20	302.25	21.73	95.40
2,7,8,9-Tricyclazole	C_9_H_7_N_3_S	−0.13	189.04	12.13	96.60
2-Amino-1,3,4-octadecanetriol	C_18_H_39_NO_3_	−0.12	317.29	17.35	80.30
2-Amino-4-methylpyrimidine	C_5_H_7_N_3_	0.28	109.06	1.03	59.90
2-Hydroxybenzothiazole	C_7_H_5_NOS	0.02	151.01	11.79	96.60
3-(2,6-Dioxocyclohexyl)propanenitrile	C_9_H_11_NO_2_	0.29	165.08	8.53	50.10
3-(2-methylpropyl)-octahydropyrrolo[1,2-a]pyrazine-1,4-dione	C_11_H_18_N_2_O_2_	0.29	210.14	10.14	83.90
3,5-Dihydroxy-2-(4-hydroxyphenyl)-4-oxo-3,4-dihydro-2H-chromen-7-yl hexopyranoside	C_21_H_22_O_11_	0.24	450.12	9.31	78.80
3,5-di-tert-Butyl-4-hydroxybenzaldehyde	C_15_H_22_O_2_	0.37	234.16	18.81	98.40
3-amino-5-(thien-2-yl)thiophene-2-carboxamide	C_10_H_9_NOS_2_	−0.12	223.01	15.03	66.20
3-Hydroxy-2-methylpyridine	C_6_H_7_NO	0.24	109.05	1.27	65.30
3-oxoindane-1-carboxylic acid	C_10_H_8_O_3_	0.03	176.05	15.64	61.30
4-[2-cyano-2-(2-pyridyl)vinyl]phenyl thiophene-2-carboxylate	C_19_H_12_N_2_O_2_S	−0.03	332.06	1.00	84.20
4-hydroxy-6,9-dimethyl-3-methylidene-2H,3H,3aH,4H,5H,6H,6aH,7H,8H,9bH-azuleno[4,5-b]furan-2,8-dione	C_15_H_18_O_4_	0.43	262.12	9.95	71.00
5-hydroxy-4-methoxy-5,6-dihydro-2H-pyran-2-one	C_6_H_8_O_4_	0.07	144.04	15.44	59.40
5-Hydroxymethyl-2-furaldehyde	C_6_H_6_O_3_	0.00	126.03	2.31	99.40
6-Pentyl-2H-pyran-2-one	C_10_H_14_O_2_	0.38	166.10	12.74	57.30
9S,13R-12-Oxophytodienoic acid	C_18_H_28_O_3_	−0.28	292.20	16.56	91.00
Adenosine	C_10_H_13_N_5_O_4_	−4.67	267.10	1.72	97.20
Afzelin	C_21_H_20_O_10_	−0.21	432.11	14.06	97.00
Amiodarone	C_25_H_29_I_2_NO_3_	0.36	645.02	18.74	94.10
Apocynin	C_9_H_10_O_3_	0.16	166.06	1.75	53.90
Arecoline	C_8_ H_13_ N O_2_	0.25	155.09	1.26	77.70
Asparagine	C_4_H_8_N_2_O_3_	−0.06	132.05	1.11	88.50
Azithromycin	C_38_H_72_N_2_O_12_	−0.49	748.51	12.23	96.00
Azoxystrobin	C_22_H_17_N_3_O_5_	−0.11	403.12	16.63	60.80
Benzothiazole	C_7_H_5_NS	0.44	135.01	12.04	89.50
Bis(4-ethylbenzylidene)sorbitol	C_24_H_30_O_6_	0.41	414.20	17.99	98.50
Bromhexine	C_14_H_20_Br_2_N_2_	−0.02	374.00	13.69	86.60
Butyl 4-aminobenzoate	C_11_H_15_NO_2_	0.39	193.11	13.25	92.10
Carbamazepine	C_15_H_12_N_2_O	−0.14	236.09	14.48	99.50
Cetrimonium	C_19_H_41_N	0.22	283.32	19.93	94.90
Choline	C_5_H_13_NO	−0.63	103.10	1.04	95.40
D-Glucosamine	C_6_H_13_NO_5_	−0.56	179.08	0.91	58.60
Dibenzylamine	C_14_H_15_N	0.18	197.12	9.83	66.00
Diethyl phthalate	C_12_H_14_O_4_	−0.41	222.09	15.46	99.80
Difenoconazole	C_19_H_17_Cl_2_N_3_O_3_	0.04	405.06	19.38	84.20
Diisobutylphthalate	C_16_H_22_O_4_	0.34	278.15	15.31	45.70
DL-2-(acetylamino)-3-phenylpropanoic acid	C_11_H_13_NO_3_	0.28	207.09	12.09	40.80
DL-Arginine	C_6_H_14_N_4_O_2_	0.31	174.11	0.98	90.10
DL-Tryptophan	C_11_H_12_N_2_O_2_	0.59	204.09	3.81	94.60
Dodine	C_13_H_29_N_3_	0.26	227.24	18.11	97.20
Erucamide	C_22_H_43_NO	−0.56	337.33	23.25	86.40
Genistein	C_15_H_10_O_5_	−0.27	270.05	9.12	79.20
Genistin	C_21_H_20_O_10_	−0.02	432.11	10.02	95.60
Glycyl-L-leucine	C_8_H_16_N_2_O_3_	−0.01	188.12	1.53	48.40
Griseofulvin	C_17_H_17_ClO_6_	−0.56	352.07	15.63	96.60
Hydroxyprogesterone caproate	C_27_H_40_O_4_	0.69	428.29	20.55	73.60
Hypoxanthine	C_5_H_4_N_4_O	−0.08	136.04	1.29	58.50
Iminostilbene	C_14_H_11_N	0.52	193.09	14.48	94.10
Indole-3-acetyl-L-aspartic acid	C_14_H_14_N_2_O_5_	−0.36	290.09	10.26	43.50
Irbesartan	C_25_H_28_N_6_O	0.45	428.23	16.03	95.30
Isoamylamine	C_5_H_13_N	−0.09	87.10	14.89	45.90
Isobutyraldehyde	C_4_H_8_O	0.70	72.06	3.38	82.90
Isoleucine	C_6_H_13_NO_2_	−0.29	131.09	2.28	96.80
Isophorone	C_9_H_14_O	0.32	138.10	8.23	94.80
Isoquinoline	C_9_H_7_N	0.46	129.06	10.28	76.20
Kaempferol	C_15_H_10_O_6_	−0.50	286.05	14.06	99.80
Kynurenic acid	C_10_H_7_NO_3_	0.38	189.04	7.53	99.20
L-Aspartic acid	C_4_H_7_NO_4_	0.35	133.04	1.13	74.00
Leucylproline	C_11_H_20_N_2_O_3_	0.31	228.15	1.87	82.80
L-Histidine	C_6_ H_9_ N_3_O_2_	−0.11	155.07	0.89	96.60
Linoleoyl Ethanolamide	C_20_H_37_NO_2_	0.18	323.28	21.97	70.20
L-Methionine sulfoxide	C_5_H_11_NO_3_S	0.54	165.05	1.07	69.70
L-Norleucine	C_6_H_13_NO_2_	−0.27	131.09	1.41	99.30
L-Phenylalanine	C_9_H_11_NO_2_	0.29	165.08	2.17	98.20
L-Threonine	C_4_H_9_NO_3_	0.27	119.06	1.11	62.30
L-Tyrosine	C_9_H_11_NO_3_	−0.05	181.07	1.34	63.30
L-Valine	C_5_H_11_NO_2_	−0.04	117.08	1.86	96.20
Metalaxyl	C_15_H_21_NO_4_	−0.34	279.15	15.41	95.40
Minoxidil	C_9_ H_15_ N_5_ O	0.86	209.13	8.89	90.40
Monoolein	C_21_H_40_O_4_	−0.91	356.29	22.89	95.50
Muscone	C_16_H_30_O	−0.56	238.23	22.63	59.50
Myricetin	C_15_H_10_O_8_	−0.62	318.04	12.11	99.30
Myricitrin	C_21_H_20_O_12_	0.01	464.10	12.07	92.50
N,N-Diisopropylethylamine (DIPEA)	C_8_H_19_N	0.13	129.15	5.78	53.40
N-[4-(6-methyl-1,3-benzothiazol-2-yl)phenyl]benzamide	C_21_H_16_N_2_OS	0.39	344.10	0.96	60.10
N-Acetyl-DL-tryptophan	C_13_H_14_N_2_O_3_	−0.08	246.10	10.55	98.60
N-Acetylornithine	C_7_H_14_N_2_O_3_	−0.30	174.10	1.05	54.00
Neosaxitoxin	C_10_H_17_N_7_O_5_	0.74	315.13	1.53	55.40
Nicotinic acid	C_6_H_5_NO_2_	0.14	123.03	2.12	71.70
Nootkatone	C_15_H_22_O	0.19	218.17	18.85	96.80
NP-002322	C_18_H_32_O_4_	−0.38	312.23	19.02	95.50
NP-007909	C_13_ H_20_ O_3_	0.24	224.14	11.21	47.50
NP-016455	C_11_H_18_N_2_O_4_	0.92	242.13	9.04	63.10
NP-019491	C_15_H_22_O_4_	−0.11	266.15	10.90	60.80
NP-019722	C_8_H_13_NO_4_	0.66	187.08	1.11	48.80
NP-019811	C_6_H_7_NO_2_	0.06	125.05	3.02	58.90
NP-019811	C_6_H_7_NO_2_	0.12	125.05	2.73	44.70
Oleamide	C_18_H_35_NO	−0.75	281.27	22.63	94.40
Paracetamol	C_8_H_9_NO_2_	0.69	151.06	3.06	81.50
Phenacetin	C_10_H_13_NO_2_	−0.23	179.09	9.51	40.00
Pipecolic acid	C_6_H_11_NO_2_	0.61	129.08	1.14	87.10
Proline	C_5_H_9_NO_2_	0.18	115.06	1.11	98.50
Prolylleucine	C_11_H_20_N_2_O_3_	0.31	228.15	1.18	81.70
Propionylcarnitine	C_10_H_19_NO_4_	−0.41	217.13	1.43	97.90
Protirelin	C_16_H_22_N_6_O_4_	−4.32	362.17	0.98	89.20
Pyrogallol	C_6_H_6_O_3_	−0.06	126.03	8.25	82.90
Quercetin	C_15_H_10_O_7_	−0.21	302.04	13.22	99.60
Quercetin-3β-D-glucoside	C_21_H_20_O_12_	0.07	464.10	12.44	95.00
Tolycaine	C_15_H_22_N_2_O_3_	0.22	278.16	9.47	82.30
Trans-3-Indoleacrylic acid	C_11_H_9_NO_2_	0.31	187.06	10.27	95.70
Tributylamine	C_12_H_27_N	0.53	185.21	10.94	90.00
Trigonelline	C_7_H_7_NO_2_	0.39	137.05	1.76	86.00
Valine	C_5_H_11_NO_2_	−0.04	117.08	1.50	95.40
Viloxazine	C_13_H_19_NO_3_	0.19	237.14	1.85	64.20
Zaleplon	C_17_H_15_N_5_O	−4.66	305.13	12.87	55.50
α,α-Trehalose	C_12_H_22_O_11_	0.04	342.12	1.03	77.70
(+/-)9,10-dihydroxy-12Z-octadecenoic acid	C_18_H_34_O_4_	1.46	314.25	19.60	87.30
(+/-)9-HpODE	C_18_H_32_O_4_	1.06	312.23	18.99	70.70
(15Z)-9,12,13-Trihydroxy-15-octadecenoic acid	C_18_H_34_O_5_	0.37	330.24	17.23	90.90
2-(acetylamino)-3-(1H-indol-3-yl) propanoic acid	C_13_H_14_N_2_O_3_	0.27	246.10	10.26	98.50
2,4,6-Trihydroxy-2-(4-hydroxybenzyl)-1-benzofuran-3(2H)-one	C_15_H_12_O_6_	0.78	288.06	11.77	94.40
2-Furoic acid	C_5_H_4_O_3_	1.01	112.02	1.28	58.30
3,5-Dihydroxy-2-(4-hydroxyphenyl)-4-oxo-3,4-dihydro-2H-chromen-7-yl hexopyranoside	C_21_H_22_O_11_	1.11	450.12	9.33	78.80
5-Aminovaleric acid	C_5_H_11_NO_2_	1.10	117.08	1.07	71.20
Acrylic acid	C_3_H_4_O_2_	0.97	72.02	1.03	46.10
Afzelin	C_21_H_20_O_10_	0.26	432.11	14.07	95.20
Asparagine	C_4_H_8_N_2_O_3_	0.84	132.05	0.99	60.70
Citric acid	C_6_H_8_O_7_	0.89	192.03	1.29	99.20
Corchorifatty acid F	C_18_H_32_O_5_	0.75	328.23	16.56	89.80
D-(-)-Fructose	C_6_H_12_O_6_	0.78	180.06	2.91	98.60
D-(+)-Galactose	C_6_H_12_O_6_	0.77	180.06	33.15	94.40
DL-Lactic Acid	C_3_H_6_O_3_	0.77	90.03	1.30	87.90
DL-Malic acid	C_4_H_6_O_5_	1.05	134.02	1.11	99.10
DL-β-Leucine	C_6_H_13_NO_2_	0.73	131.09	1.51	97.40
Dodecyl sulfate	C_12_H_26_O_4_ S	0.75	266.16	24.13	96.00
D-Saccharic acid	C_6_H_10_O_8_	0.72	210.04	1.13	59.80
Flutamide	C_11_H_11_F_3_N_2_O_3_	1.01	276.07	16.72	97.50
Gentisic acid	C_7_H_6_O_4_	1.29	154.03	2.96	94.40
Gluconic acid	C_6_H_12_O_7_	0.39	196.06	1.10	97.70
L-Histidine	C_6_H_9_N_3_O_2_	0.75	155.07	0.98	98.40
Myricitrin	C_21_H_20_O_12_	1.37	464.10	12.11	97.60
Trans-Aconitic acid	C_6_H_6_O_6_	0.32	174.02	1.17	73.20

**Figure 3:**
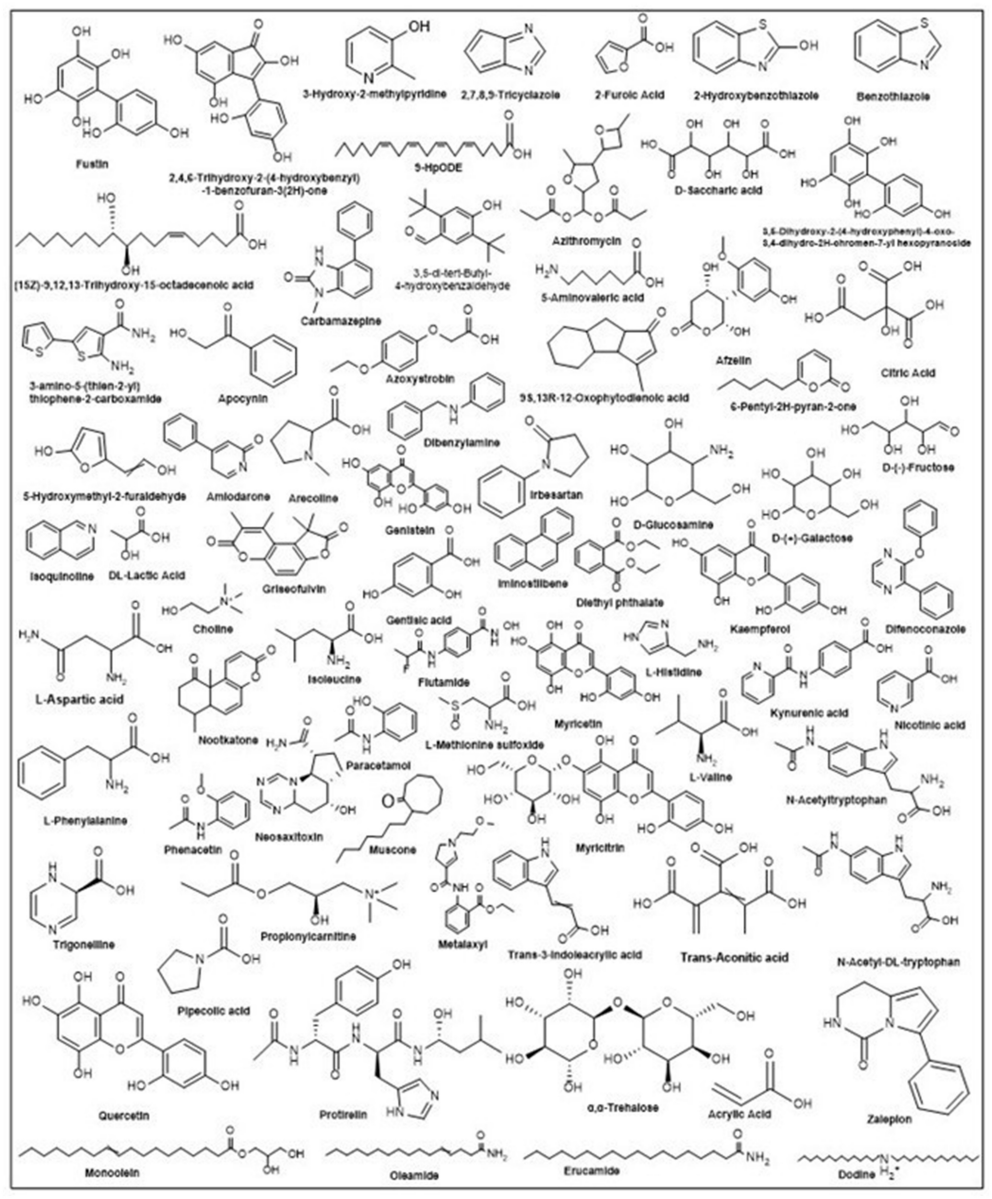
Structures of identified bioactive compounds and their structures observed in the aril of Manila tamarind.

Various phytochemicals identified in Manila tamarind (Table 4) were antioxidant activity (25), anti-inflammatory activity (22), anticancer activity (15), neuroprotective activity (13), antifungal activity (11), antibacterial activity (11), antimicrobial activity (10), antiviral activity (10), anti-tumor activity (6), analgesic activity (6), cardio-protective activity (5), hepatoprotective activity (4), anticonvulsant activity (4), antidiabetic activity (3), anesthetic activity (3), sedative activity (3), anti-ulcer activity (2), antiseptic activity (2), antipyretic activity (2), anthelmintic activity (2), antimalarial activity (2), nematocidal activity (2), antimigraine activity (1), antiarrhythmic activity (1), anti-amyloidogenic activity (1), antiandrogen activity (1), antifibrotic activity (1), osteoarthritis activity (1), angiogenesis activity (1), antifeedant activity (1), anti-epileptic activity (1), anticerebral ischemia activity (1), anti-leishmanial activity (1), anti-allergic activity (1), atherosclerotic activity (1), anti-stress activity (1), antirheumatic activity (1), antinociceptive activity (1), anti-cardiovascular activity (1), anti-mineralocorticoid activity (1), anti-parasitic activity (1), antidementia activity (1), and anti-cerebrovascular activity (1). The chemical structures of the representative compounds are illustrated in Figure 3.

**Table 4:** Categorization of bioactive compounds (Phytochemicals) identified in Manila tamarind based on medicinal properties.

**S. No**.	**Medicinal properties**	**Mode of analysis**	**Bioactive compounds**
1	Anti-inflammatory	PM	Afzelin, Apocynin, Azithromycin, Benzothiazole, Choline, Genistein, Griseofulvin, Isoquinoline, Kaempferol, L-Methionine sulfoxide, L-Valine, 9S, 13R-12-Oxophytodienoic acid, Monoolein, Muscone, N-Acetyl-DL-tryptophan, Myricitrin, Nicotinic acid, Nootkatone, Quercetin, Trans-Aconitic Acid, 3,5-Dihydroxy-2-(4-hydroxyphenyl)-4-oxo-3,4-dihydro-2H-chromen-7-yl hexopyranoside, 3,5-di-tert-Butyl-4-hydroxybenzaldehyde
		NM	Afzelin, Trans-Aconitic acid
2	Antioxidant	PM	2,4,6-Trihydroxy-2-(4-hydroxybenzyl)-1-benzofuran-3(2H)-one, 3,5-Dihydroxy-2-(4-hydroxyphenyl)-4-oxo-3,4-dihydro-2H-chromen-7-yl hexopyranoside, 3,5-di-tert-Butyl-4-hydroxybenzaldehyde, 3-amino-5-(thien-2-yl)thiophene-2-carboxamide, 5-Hydroxymethyl-2-furaldehyde, Afzelin, Benzothiazole, Citric Acid, Dibenzylamine, Genistein, Azoxystrobin, Gentisic Acid, Irbesartan, Isoquinoline, Kaempferol, L-Histidine, Myricetin, N-Acetyltryptophan, Nootkatone, Quercetin, Valine
		NM	Afzelin, Citric acid, Corchorifatty acid F, D-(-)-Fructose, D-(+)-Galactose, D-Saccharic acid, Gentisic acid, L-Histidine, Myricitrin
3	Neuroprotective	PM	Afzelin, Choline, Dibenzylamine, Diethyl phthalate, Kaempferol, Kynurenic acid, L-Phenylalani, Muscone, N-Acetyl-DL-tryptophan, Nootkatone, L-Aspartic acid, Protirelin, Trigonelline
		NM	Gentisic acid
4	Anticancer	PM	2-Hydroxybenzothiazole, Afzelin, Arecoline, Benzothiazole, Dibenzylamine, D-Saccharic Acid, Flutamide (prostate cancer), Isoleucine, Isoquinoline, Kaempferol, Monoolein, Myricetin, Nootkatone, Trigonelline
		NM	Gentisic acid, 5-Aminovaleric acid
5	Antiviral	PM	2-Hydroxybenzothiazole, Benzothiazole, Genistein, Genistin, Griseofulvin, Isoquinoline, Myricetin, Quercetin, L-Valine, Trigonelline
6	Antibacterial	PM	Azithromycin, Benzothiazole, Genistein, Isoquinoline, Myricetin, Nootkatone, Pipecolic acid, Trans-3-Indoleacrylic acid, Trigonelline
		NM	Citric acid, DL-Lactic Acid, (+/-)9-HpODE
7	Antifungal	PM	2,7,8,9-Tricyclazole, 6-Pentyl-2H-pyran-2-one, Azoxystrobin, Difenoconazole, Dodine, Griseofulvin, Metalaxyl, Trans-3-Indoleacrylic acid
		NM	(15Z)-9,12,13-Trihydroxy-15-octadecenoic acid, Corchorifatty acid F, (+/-)9-HpODE, Trans-Aconitic acid
8	Anti-tumor	PM	3-Hydroxy-2-methylpyridine, Benzothiazole, D-Glucosamine, Griseofulvin, Monoolein, Trigonelline
9	Analgesic	PM	3,5-di-tert-Butyl-4-hydroxybenzaldehyde, L-Valine, Paracetamol, Phenacetin, Propionylcarnitine
		NM	Gentisic acid
10	Anticonvulsant	PM	2-Hydroxybenzothiazole, Benzothiazole, Carbamazepine, Kynurenic acid
11	Antimicrobial	PM	2-Furoic Acid, 2-Hydroxybenzothiazole, Acrylic Acid, Bis (4-ethylbenzylidene) sorbitol, Diethyl phthalate, Myricitrin, Quercetin
		NM	Acrylic acid, Citric acid, Gentisic acid
12	Cardioprotective	PM	Choline, Kaempferol, Muscone, Nootkatone
		NM	Gentisic acid
13	Hepatoprotective	PM	Nootkatone, Quercetin, 5-Hydroxymethyl-2-furaldehyde
		NM	Gentisic acid
14	Sedative	PM	Oleamide, Quercetin, Trigonelline, Zaleplon
15	Antidiabetic	PM	Isoquinoline, Kaempferol, Quercetin
16	Anesthetic	PM	Butyl 4-aminobenzoate, Neosaxitoxin
17	Antiseptic	PM	Cetrimonium (Topical antiseptic), Quercetin
18	Antipyretic	PM	Paracetamol, Phenacetin
19	Anthelmintic	PM	Benzothiazole, Arecoline
20	Antimalarial	PM	Benzothiazole, 2-Hydroxybenzothiazole
21	Anti-ulcer	PM	Myricetin, (-)-Fustin
22	Nematocidal	NM	Trans-Aconitic acid, 2-Furoic acid
23	Anti mineralocorticoid	PM	Hydroxyprogesterone caproate
24	Antimigraine	PM	Trigonelline
25	Antiarrhythmic agent	PM	Amiodarone
26	Antinociceptive	PM	Nicotinic acid
27	Anti-amyloidogenic, Anti-epileptic	PM	Myricetin
28	Anti-cerebrovascular and Anticardiovascular	PM	Iminostilbene
29	Antiandrogen	NM	Flutamide
30	Antifibrotic	PM	Nootkatone
31	Antirheumatic	PM	Propionylcarnitine
32	Osteoarthritis	PM	D-Glucosamine
33	Angiogenesis	PM	Erucamide
34	Antidementia and Anticerebral ischemia	PM	Muscone
35	Anti-allergic	PM	Kaempferol
36	Anti-parasitic	PM	Arecoline
37	Atherosclerotic Effects	NM	(15Z)-9,12,13-Trihydroxy-15-octadecenoic acid
38	Anti-stress	NM	α,α-Trehalose
39	Antifeedant and Anti-Leishmanial Activity	NM	Trans-Aconitic acid

PM, Positive mode of analysis; NM, Negative mode of analysis.

Early detection, improved treatment options, and preventive measures, such as healthy diets, remain critical strategies to combat this growing health challenge (4, 5). In Manila tamarind, 16 anti-cancerous compounds (Table 4) were identified through positive mode (2-hydroxybenzothiazole, afzelin, arecoline, benzothiazole, dibenzylamine, d-saccharic acid, flutamide (prostate cancer), isoleucine, isoquinoline, kaempferol, monoolein, myricetin, nootkatone, trigonelline) and negative mode of analysis (gentisic acid and 5-aminovaleric acid). These identified phytochemicals control different types of cancer such as breast cancer (2-hydroxybenzothiazole, afzelin, benzothiazole, kaempferol, myricetin, and trigonelline), colon cancer (D-saccharic acid, afzelin, myricetin, nootkatone, and gentisic acid), prostate cancer (flutamide), liver cancer (nootkatone and trigonelline), leukemia (isoquinoline derivatives), and pancreatic cancer (kaempferol and myricetin).

Consuming a diet rich in antioxidants from fruits, vegetables, and phytochemicals has been linked to a lower risk of many serious health conditions, such as cancer (14), cardiovascular diseases (25), diabetes mellitus (26), neurodegenerative diseases such as Alzheimer's, Parkinson's (27), inflammatory diseases (28), aging (29), kidney disorders (30), liver diseases (31), skin disorders (32), and respiratory diseases, e.g., COPD and asthma (33).

#### 3.4.1 Alkaloids

Alkaloids are a diverse group of nitrogen-containing secondary metabolites found in plants, fungi, and certain animal species, which are known for their potent pharmacological properties. These compounds play a critical role in the development of modern pharmaceuticals, ranging from analgesics to chemotherapeutic agents (34). In Manila tamarind, we identified 18 alkaloid compounds, viz., arecoline, isoquinoline, azithromycin, muscone, trigonelline, isoamylamine, azoxystrobin, L-tyrosine, L-histidine, L-phenylalanine, L-norleucine, DL-tryptophan, N-acetyl-DL-tryptophan, DL-arginine, proline, propionylcarnitine, leucylproline, and prolylleucine, which are often used for several health benefits (Tables 4, 5). Historically, alkaloids like morphine and its derivative codeine have been essential in pain management, while quinine revolutionized malaria treatment and offers anti-inflammatory effects (35). Quinine was the first effective treatment for malaria and is used in the treatment of resistant strains of *Plasmodium falciparum*, and also possesses analgesic and anti-inflammatory properties (36). Caffeine, a methylxanthine alkaloid, stimulates the central nervous system and is linked to reduced risks of Parkinson's and Alzheimer's disease (37). Despite its addictive nature, nicotine from *Nicotiana tabacum* shows neuroprotective potential and is used in smoking cessation therapies (38). Ephedrine has long been used as a bronchodilator and nasal decongestant. It stimulates adrenergic receptors, leading to increased heart rate and bronchial relaxation (39). Atropine, used in clinical settings to dilate pupils, treat bradycardia, and counteract the effects of organophosphate poisoning (40). Reserpine was among the first alkaloids used to manage hypertension and certain psychiatric disorders. It depletes catecholamines and serotonin from central and peripheral neurons, which explains its tranquilizing effect (41). Berberine, a protoberberine alkaloid found in *Berberis* species, has gained attention for its antimicrobial, anti-inflammatory, and antidiabetic properties. It has been shown to lower blood glucose and cholesterol levels, making it a promising agent for metabolic syndrome (42). In oncology, vincristine and vinblastine, derived from the *Catharanthus roseus* plant, are widely used chemotherapeutic agents. These vinca alkaloids inhibit mitosis by binding to tubulin and are essential in treating leukemias, lymphomas, and other cancers (43). Though strychnine, an alkaloid from *Strychnos nux-vomica*, is primarily known for its toxicity, it has historically been used in small doses as a stimulant and performance enhancer. Its mechanism involves the inhibition of glycine receptors, which affects motor neurons (44). Yohimbine, derived from *Pausinystalia yohimbe*, has been used for erectile dysfunction and is being studied for potential benefits in weight loss and anxiety treatment (45). The presence of these bioactive alkaloids in Manila tamarind highlights their significant therapeutic potential. Continued research is warranted to better understand their mechanisms of action and broaden their applications in clinical medicine.

**Table 5:** Categorization of phytochemicals of different Manila tamarind accessions based on the classes.

**S.N**.	**Categories**	**Phytochemical**
1	Alkaloids	Arecoline, Isoquinoline, Azithromycin, Muscone, Trigonelline, Isoamylamine, Azoxystrobin, L-Tyrosine, L-Histidine, L-Phenylalanine, L-Norleucine, DL-Tryptophan, N-Acetyl-DL-tryptophan, DL-Arginine, Proline, Propionylcarnitine, Leucylproline, and Prolylleucine
2	Flavonoids	Kaempferol, Quercetin, Quercetin-3β-D-glucoside, Myricitrin, Myricetin, (-)-Fustin, Afzelin, Genistin, and Genistein
3	Phenolics and polyphenols	Pyrogallol, Gentisic acid, 5-Hydroxymethyl-2-furaldehyde, Corchorifatty acid F, Hydroxyprogesterone caproate, Apocynin, Linoleoyl Ethanolamide, 2,4,6-Trihydroxy-2-(4-hydroxy benzyl)-1-benzofuran-3(2H)-one, and (2E)-3-(4-{[(2S,3R,4S,5S,6R)-3,4,5-trihydroxy-6-(hydroxymethyl) oxan-2-yl]oxy}phenyl)prop-2-enoic acid
4	Amino acids and derivatives	Choline, L-valine, Valine, Asparagine, L-threonine, L-aspartic acid, 5-amino valeric acid, DL-β-Leucine, and 2-Amino-4-methyl pyrimidine
5	Carbohydrates and sugars	D-(+)-Galactose, D-(+)-Glucose, D-(-)-Fructose, α,α-Trehalose, DL-Lactic Acid, D-Saccharic acid, Gluconic acid, and D-Glucosamine
6	Fatty acids and lipids	1-Stearoylglycerol, Monoolein, 2-Amino-1,3,4-octadecanetriol, Butyl 4-aminobenzoate, Diethyl phthalate, Di-isobutylphthalate, and Bis (4-ethylbenzylidene) sorbitol
7	Terpenes and terpenoids	Nootkatone, Difenoconazole, 6-Pentyl-2H-pyran-2-one, 9S,13R-12-Oxophytodienoic acid, (±)-Abscisic acid, (15Z)-9,12,13-Trihydroxy-15-octadecenoic acid, (+/-)9-HpODE, and (+/-)9,10-dihydroxy-12Z-octadecenoic acid
8	Organic acids and derivatives	Citric acid, DL-Malic acid, acrylic acid, 2-Furoic acid, trans-aconitic acid, 2-(acetylamino)-3-(1H-indol-3-yl) propanoic acid
9	Steroids and hormones	Paracetamol, Flutamide, Bromhexine,3,5-Dihydroxy-2-(4-hydroxyphenyl)-4-oxo-3,4-dihydro-2H-chromen-7-yl hexopyranoside, Irbesartan, Viloxazine, and Amiodarone
10	Pyridine and pyrimidine compounds	Nicotinic acid, 3-Hydroxy-2-methylpyridine, Kynurenic acid, and (3R,4S)-1-(4-Morpholinylcarbonyl)-3-(2-{4-[3-(trifluoromethyl) phenyl]-1-piperazinyl} ethyl)-4-piperidinyl] acetic acid and Cetrimonium
11	Heterocyclic compounds	Isophorone, Erucamide, NP-019811, NP-019722, NP-019491, NP-002322, NP-007909, NP-016455, NP-019811, and NP-019491
12	Miscellaneous	Dodine, Metalaxyl, Griseofulvin, 3-oxoindane-1-carboxylic acid, 2,3-dihydroxypropyl 12-methyltridecanoate, 5-hydroxy-4-methoxy-5,6-dihydro-2H-pyran-2-one, (2S6′R)2′,4,6-trimethoxy6′-methyl-3H-spiro[1-benzofuran-21′-cyclohexan]2′-ene-34′-dione, 1-(3,4-dimethoxyphenyl) ethan-1-one oxime, Dibenzylamine, 2,7,8,9-Tricyclazole, Tolycaine, trans-3-Indoleacrylic acid, 3,5-di-tert-Butyl-4-hydroxybenzaldehyde, 1-(2-Morpholinophenyl)dihydro-1H-pyrrole-2,5-dione, 3-(2-methylpropyl)-octahydropyrrolo [1,2-a]pyrazine-1,4-dione, 3-(2,6-Dioxocyclohexyl) propanenitrile, Iminostilbene, Zaleplon, 3-amino-5-(thien-2-yl)thiophene-2-carboxamide, L-Methionine sulfoxide, Carbamazepine, Adenosine, Phenacetin, Isobutyraldehyde, Tributylamine, Dodecyl sulfate, Benzothiazole, 2-Hydroxybenzothiazole, N,N-Diisopropylethylamine (DIPEA), N-Acetylornithine, Oleamide, and N-[4-(6-methyl-1,3-benzothiazol-2-yl) phenyl] benzamide

#### 3.4.2 Flavonoids

Flavonoids are another prevalent class of polyphenolic compounds found in fruits, vegetables, and medicinal plants, renowned for their antioxidant, anti-inflammatory, anticancer, and cardioprotective properties. The phytochemical screening resulted in nine flavonoid compounds (kaempferol, quercetin, quercetin-3β-D-glucoside, myricitrin, myricetin, (-)-fustin, afzelin, genistin, and genistein) in the aril of Manila tamarind (Table 5). Among them, kaempferol and quercetin have been extensively studied for their health-promoting effects. Kaempferol exhibits strong antioxidant and anti-inflammatory activities and has been shown to inhibit cancer cell proliferation and induce apoptosis, particularly in breast and liver cancer models (46). Quercetin, another abundant flavonol, contributes to cardiovascular health by reducing oxidative stress, lowering blood pressure, and improving endothelial function (47). Its glycosylated derivative, quercetin-3β-D-glucoside, shows significant bioavailability and exhibits similar biological activities, including anti-diabetic and neuroprotective effects (48). Myricetin and its glycoside myricitrin also exhibit significant bioactivity. Myricetin has demonstrated anticancer effects via modulation of key signaling pathways such as PI3K/Akt and MAPK, while myricitrin exerts hepatoprotective and anti-inflammatory actions (49). The flavonol (-)-fustin, found primarily in *Rhus* species, has antioxidant and antidiabetic effects, attributed to its ability to inhibit aldose reductase and prevent lipid peroxidation (50). Similarly, afzelin, a kaempferol glycoside, has shown anti-inflammatory, anti-allergic, and anticancer properties through the inhibition of mast cell degranulation and NF-κB activation (51). Isoflavones such as genistin and genistein, predominantly found in soy products, have been widely explored for their phytoestrogenic activity. Genistein, in particular, exhibits potent anticancer, antioxidant, and osteoprotective properties and is being investigated for its role in hormone-related cancers and osteoporosis prevention (52). Genistin, the glycosylated form of genistein, is more water-soluble and converts to the active aglycone in the gut, making it effective in delivering systemic benefits, including cardiovascular and bone health support (53). Collectively, the flavonoids identified in Manila tamarind present diverse therapeutic potential and are promising candidates for natural drug development and preventive healthcare. Further studies are needed to optimize their bioavailability and substantiate their clinical efficacy.

#### 3.4.3 Phenolics and polyphenols

Phenols and polyphenols are plant-derived secondary metabolites with notable anti-inflammatory, anticancer, antioxidant, and antibacterial properties. Phytochemical screening of Manila tamarind aril revealed nine phenolic and polyphenolic compounds (Table 5). Among them, pyrogallol—a trihydroxybenzene derivative—displays both antioxidant and pro-oxidant effects and has shown cytotoxicity against cancer cells *via* oxidative stress mechanisms (54). Gentisic acid, a type of dihydroxybenzoic acid, is known for its anti-inflammatory and analgesic effects, primarily through modulation of the cyclooxygenase (COX) pathway and neutralization of reactive oxygen species (55). 5-Hydroxymethyl-2-furaldehyde (5-HMF), a degradation product of carbohydrates, exhibits notable antioxidant, anti-sickle cell, and cytoprotective activities. It interacts with hemoglobin to increase oxygen affinity and has demonstrated therapeutic potential in preclinical models of cardiovascular disorders (56).

Corchorifatty acid F, a lesser-known polyunsaturated fatty acid derivative from *Corchorus* species, has been preliminarily noted for its anti-inflammatory and lipid-modulating potential (57). Hydroxyprogesterone caproate (HPC), a synthetic progestin primarily used to prevent preterm birth, also demonstrates anti-inflammatory activity by engaging glucocorticoid receptor-mediated pathways and regulating cytokine expression (58). Apocynin, a methoxy-substituted catechol, is recognized for its ability to inhibit NADPH oxidase activity, reducing oxidative stress and inflammation, particularly in models of neurodegeneration, cardiovascular disease, and diabetes (59). Linoleoyl ethanolamide (LEA), an endogenous lipid amide, acts as a signaling molecule in energy metabolism and inflammation. It exerts anti-obesity and anti-inflammatory effects, potentially by modulating peroxisome proliferator-activated receptors (PPARs) and inhibiting pro-inflammatory cytokines (60). The compound 2,4,6-trihydroxy-2-(4-hydroxybenzyl)-1-benzofuran-3(2H)-one, structurally related to aurones, has shown strong antioxidant and anti-tyrosinase activity, making it a candidate for skin-whitening and neuroprotective therapies (https://www.pubchem.ncbi.nlm.nih.gov/compound/54378453). The (2E)-3-(4-{[(2S,3R,4S,5S,6R)-3,4,5-trihydroxy-6-(hydroxymethyl) oxan-2-yl]oxy}phenyl)prop-2-enoic acid, a glycosylated derivative of *p*-coumaric acid, combines antioxidant and hepatoprotective properties, and may play a role in modulating glucose and lipid metabolism in metabolic syndrome (61). These phenolic and polyphenolic compounds identified in Manila tamarind collectively demonstrate promising bioactivities that justify further exploration as therapeutic agents or dietary supplements.

#### 3.4.4 Amino acids and derivatives

Amino acids and their derivatives are essential for a wide range of physiological processes, including protein synthesis, neurotransmission, metabolic regulation, and cellular signaling. Phytochemical screening of the aril of Manila tamarind identified nine amino acids and related compounds (Table 5). Among them, choline is an essential nutrient that supports cell membrane integrity and serves as a precursor for the neurotransmitter acetylcholine. It also plays a key role in methyl group metabolism and exhibits both neuroprotective and hepatoprotective properties (62). L-valine and valine, branched-chain amino acids (BCAAs), play critical roles in muscle metabolism and energy production, particularly during exercise and catabolic stress. They enhance muscle repair and reduce exercise-induced fatigue (63). Similarly, asparagine is involved in nitrogen transport and protein glycosylation, and its availability has been linked to cancer cell adaptability and metastasis (64). L-threonine, a key component of mucin proteins, contributes to intestinal health and immune modulation, particularly in neonatal and weaning animals (65). L-aspartic acid functions as both a building block for protein synthesis and a neurotransmitter in the central nervous system (66). 5-Aminovaleric acid, a GABA analog, has shown potential in modulating neuroactive signaling and holds promise for therapeutic intervention in neurodegenerative disorders (67). DL-β-Leucine may exert modulatory effects on neurotransmission and serve as a precursor in synthetic biochemical pathways (68). Finally, 2-Amino-4-methyl pyrimidine is of pharmaceutical interest due to its structural similarity to vitamin B1 (thiamine) and potential antimicrobial and enzyme inhibitory activities (69). Collectively, these amino acids and derivatives contribute to a broad spectrum of metabolic and therapeutic functions, underscoring their significance in nutritional science and biomedical research.

#### 3.4.5 Carbohydrates and sugars

Carbohydrates are essential biomolecules that function as primary energy sources and structural components in living organisms. Phytochemical screening of the aril of Manila tamarind identified eight carbohydrate- and sugar-related compounds (Table 5). Among them, *D-(*+*)-Glucose* and *D-(-)-Fructose* are key monosaccharides involved in glycolysis and energy metabolism, contributing to ATP production and cellular respiration (70). *D-(*+*)-Galactose* is vital in the biosynthesis of glycoproteins and glycolipids, particularly in neuronal development (71). α,α*-Trehalose*, a disaccharide, functions as a stress protectant in organisms by stabilizing proteins and cellular structures under desiccation or thermal stress (72). *DL-Lactic acid*, a byproduct of anaerobic metabolism, is utilized therapeutically in skin care and wound healing due to its antimicrobial and exfoliating properties (46). *D-Saccharic acid* has been investigated for its role in detoxification and potential anti-carcinogenic effects through modulation of phase II enzyme activity (73). *Gluconic acid* is known for its chelating properties and applications in the pharmaceutical and food industries. *D-Glucosamine*, an amino sugar, is a precursor in glycosaminoglycan synthesis and is widely used for joint health, particularly in osteoarthritis management (74).

#### 3.4.6 Fatty acids and lipids

Fatty acids and lipid derivatives are essential for membrane structure, signal transduction, and energy metabolism. Phytochemical screening of *Manila tamarind* (*Pithecellobium dulce*) aril identified seven lipid-related compounds (Table 5). 1-Stearoylglycerol and monoolein, both monoacylglycerols, aid in lipid digestion and are used in drug delivery systems (75). 2-Amino-1,3,4-octadecanetriol, a sphingoid base derivative, supports membrane integrity and apoptosis signaling (76). Butyl 4-aminobenzoate, a lipid-soluble anesthetic, is common in topical applications (77). Diethyl phthalate and di-isobutylphthalate, though industrial plasticizers, are concerning due to their endocrine-disrupting potential (77). In contrast, bis(4-ethylbenzylidene) sorbitol is a non-toxic polymer clarifier with biomedical potential (78). These lipid compounds exhibit diverse roles in physiology and material science.

#### 3.4.7 Terpenes and terpenoids

Terpenes and terpenoids are structurally diverse natural products known for their broad pharmacological activities. Phytochemical screening of the aril of Manila tamarind identified eight terpene- and terpenoid-related compounds (Table 5). *Nootkatone*, a sesquiterpene, exhibits insecticidal, antimicrobial, and anti-inflammatory properties (79). *Difenoconazole*, a triazole fungicide with a terpenoid backbone, exhibits potent antifungal activity by inhibiting ergosterol biosynthesis (80). *6-Pentyl-2H-pyran-2-one*, a volatile compound from fungi, demonstrates antifungal and plant growth-promoting effects (81). *9S,13R-12-Oxophytodienoic acid* and *(*±*)-Abscisic acid* are oxylipin derivatives regulating plant stress responses and are now being explored for anti-inflammatory and antitumor activities in humans (82). Hydroxyoctadecenoic acids such as *(15Z)-9,12,13-trihydroxy-15-octadecenoic acid, (*+*/-)9-HpODE*, and *(*+*/-)9,10-dihydroxy-12Z-octadecenoic acid* are lipid mediators involved in oxidative stress and have implications in cardiovascular and inflammatory diseases (83).

#### 3.4.8 Organic acids and derivatives

Organic acids are integral to cellular metabolism and often function as signaling molecules or therapeutic agents. Phytochemical screening of the aril of Manila tamarind identified six organic acids and derivative-related compounds (Table 5). Citric acid and *DL*-malic acid are key intermediates of the tricarboxylic acid (TCA) cycle, essential for cellular energy production and carbon metabolism (70). *Acrylic acid*, while primarily industrial, has antimicrobial properties and is being examined for biopolymer development. *2-Furoic acid*, derived from biomass, has been reported for its antioxidant and antimicrobial activities (84). *Trans-aconitic acid* is an inhibitor of phosphofructokinase and may regulate glycolysis under stress (85). The tryptophan derivative *2-(acetylamino)-3-(1H-indol-3-yl) propanoic acid* (N-acetyltryptophan) serves as an antioxidant and stabilizer in therapeutic protein formulations (86).

#### 3.4.9 Steroids and hormone analogs

Steroids and hormone analogs exhibit a broad spectrum of therapeutic actions. The phytochemical screening resulted in seven steroids and hormone-related compounds in the aril of Manila tamarind (Table 5). *Paracetamol* is a widely used analgesic and antipyretic with hepatic metabolism, while flutamide is an antiandrogen used in prostate cancer therapy (87). Bromhexine serves as a mucolytic agent that enhances pulmonary secretion clearance. 3, 5-Dihydroxy-2-(4-hydroxyphenyl)-4-oxo-3,4-dihydro-2H-chromen-7-yl hexopyranoside, a flavonoid glycoside, has antioxidant and estrogenic activity (88). Irbesartan, an angiotensin II receptor blocker, is effective in hypertension and diabetic nephropathy. Viloxazine is a norepinephrine reuptake inhibitor recently approved for ADHD treatment. Amiodarone, a class III antiarrhythmic, modulates potassium and calcium channels, although it poses risks of thyroid and pulmonary toxicity (89). Collectively, these compounds underscore the therapeutic relevance of steroidal and hormone-like constituents present in the plant matrix.

#### 3.4.10 Pyridine and pyrimidine compounds

Pyridine and pyrimidine derivatives are critical scaffolds in drug design due to their bioactivity. The phytochemical screening resulted in seven pyridine and pyrimidine-related compounds in the aril of Manila tamarind (Table 5). *Nicotinic acid* (vitamin B3) is a precursor of NAD+ and NADP+, crucial in redox reactions and energy metabolism (90). *3-Hydroxy-2-methylpyridine* is structurally related to vitamin B6 and exhibits neuroprotective potential. *Kynurenic acid*, a tryptophan metabolite, acts as a neuroinhibitory agent through NMDA receptor antagonism and is implicated in neurodegenerative disorders like schizophrenia (91). The synthetic compound *(3R,4S)-1-(4-Morpholinylcarbonyl)-3-(2-{4-[3-(trifluoromethyl) phenyl]-1-piperazinyl} ethyl)-[4-piperidinyl] acetic acid* (a piperidine-based derivative) and *cetrimonium*, a quaternary ammonium salt, demonstrate antimicrobial and membrane-disrupting properties, making them valuable in antiseptics and pharmaceuticals (92).

#### 3.4.11 Heterocyclic compounds

Heterocyclic compounds are structurally diverse molecules that play vital roles in pharmaceuticals, agrochemicals, and materials science. The phytochemical screening resulted in 10 heterocyclic compounds in the aril of Manila tamarind (Table 5). *Isophorone*, a cyclic ketone, is widely used as an industrial solvent and as an intermediate in the synthesis of fine chemicals. It has also exhibited antibacterial and antifungal properties (93). *Erucamide*, though primarily an amide derived from erucic acid, features a heterocyclic moiety in many functional derivatives and is known for its lubricating and anti-blocking effects in polymer films. The NP-coded compounds (*NP-019811, NP-019722*, and *NP-019491*) are likely natural product derivatives or synthetic heterocycles identified in screening databases for their bioactivity. These types of compounds often contain nitrogen, oxygen, or sulfur in their ring structures and are known to exhibit a broad spectrum of pharmacological activities, including antimicrobial, anticancer, and neuroactive properties (94). Their structural diversity, including fused ring systems and spirocyclic configurations, makes them valuable for exploring new therapeutic targets and for structure–activity relationship (SAR) studies in drug discovery.

#### 3.4.12 Miscellaneous compounds

This category encompasses a broad array of biologically active and industrially relevant compounds that do not fit neatly into traditional classes. *Dodine* and *Metalaxyl* are widely used fungicides in agriculture, known for their systemic action and inhibition of nucleic acid synthesis in plant pathogens (95). *Griseofulvin*, a natural antifungal agent, disrupts microtubule function and is used clinically against dermatophytic infections (96). Various synthetic and semi-synthetic molecules, such as *3-oxoindane-1-carboxylic acid* and *3,5-di-tert-butyl-4-hydroxybenzaldehyde*, exhibit antioxidant or anti-inflammatory properties and are used as intermediates in medicinal chemistry (97). *Zaleplon* and *Carbamazepine* are central nervous system (CNS) active drugs, used as hypnotics and antiepileptics, respectively, highlighting the pharmacological breadth within this group (98). Natural products such as *trans-3-indoleacrylic acid* and *oleamide* are associated with neuroactive and anti-inflammatory functions. Surfactants such as *Dodecyl sulfate* and bases like *N, N-Diisopropylethylamine (DIPEA)* serve crucial roles in biochemistry and organic synthesis. Additionally, bioactive sulfur- and nitrogen-containing heterocycles such as *benzothiazole* and *2-hydroxybenzothiazole* are investigated for anticancer, antimicrobial, and enzyme-inhibitory activities (99). This chemically eclectic group underscores the importance of structural diversity in modulating biological function and facilitating innovation across pharmacology, agriculture, and industrial chemistry.

## 4 Conclusion

The increasing global emphasis on medicinally valuable fruit-bearing plants and the continuous pursuit of novel bioactive compounds provided the rationale for investigating the aril of Manila tamarind fruit pods. This study offers scientific validation of the antioxidant potential of the Manila tamarind plant, which is of considerable traditional significance. The analysis unveiled a rich spectrum of bioactive constituents with promising therapeutic applications in treating various human ailments. These findings underscore the Manila tamarind's value as a potent source of nutraceutical and pharmacological agents. To realize its full medicinal and dietary value, future studies should focus on isolating and characterizing key compounds and evaluating their biological activities through *in vitro* and *in vivo* models. Advances in metabolomics and molecular docking can further elucidate mechanisms of action and compound interactions. Such efforts could lead to the development of *Manila tamarind*-based interventions for oxidative stress, inflammation, and metabolic disorders.

## Data Availability

The original contributions presented in the study are publicly available. This data can be found here: https://figshare.com/s/15ccc0df782eb51546f5?file=57133391.
